# Art and Madness: The role of psychopathology in creativity

**DOI:** 10.1192/j.eurpsy.2025.1870

**Published:** 2025-08-26

**Authors:** F. M. Santos, P. Coelho, P. Jorge, A. Silva, R. Lopes, V. Melo, C. Rodrigues, C. Matias, T. Vieira, L. Delgado

**Affiliations:** 1 ULS Médio Tejo, Tomar, Portugal

## Abstract

**Introduction:**

It has long been believed that a correlation exists between mental illness and artistic ability. This idea dates to antiquity, with Aristotle famously stating, “No great mind has existed without a touch of madness”. Recent studies suggest that writers and artists show a higher incidence of depressive disorders (DD) and bipolar disorder (BD) compared to the general population. However, we know that not all artists suffer from mental illness, and most individuals with mental illness are neither artists nor creative geniuses. Thus, numerous questions emerge regarding the true relationship between psychopathology and creativity.

**Objectives:**

In this study, we sought to explore the correlation between psychopathology and creativity.

**Methods:**

In this literature review, research articles on the relationship between creativity and psychopathology are presented.

**Results:**

There is evidence that the genetic predisposition to BD, as well as milder forms of the disorder, is associated with increased creativity. A meta-analysis of 28 studies found a significant positive correlation between the risk of bipolar disorder and creativity (r = 0.224). Mild to moderate manifestations of the disorder can often serve as a source of inspiration, driving creative work. However, this creativity only flourishes when individuals are able to channel their emotional instability and cognitive disorganization into coherent and productive forms. In contrast, severe forms of the disorder tend to inhibit creativity, largely due to impaired concentration and disorganized thinking. Regarding DD, the research presents mixed findings. Some studies suggest a link between depression and lower creativity. A meta-analysis of 39 studies found a weak negative correlation between depressive mood and creativity (r = -0.064). However, other researchers argue that both positive and negative emotions can fuel artistic creativity. The Dual Pathway to Creativity Model supports this view, proposing that creativity—defined as the generation of original and valuable ideas—arises from cognitive flexibility and cognitive persistence. According to this model, dispositional or situational factors can influence creativity by enhancing either flexibility, persistence, or both. Negative mood states, for example, may enhance creativity by fostering persistence.

**Conclusions:**

The relationship between psychopathology and creativity is complex. A genetic predisposition to BD, especially in milder forms, may enhance creativity, but severe cases often impair it due to cognitive difficulties. For DD, the link to creativity is more conflicted. While depression is generally associated with reduced creativity, some models suggest both positive and negative emotions, including those from depressive states, can foster creativity by affecting cognitive flexibility and persistence. In summary, the connection between psychopathology and creativity is multifaceted, with various contributing factors.

**Disclosure of Interest:**

None Declared

